# Multidimensional Energy Poverty and Mental Health: Micro-Level Evidence from Ghana

**DOI:** 10.3390/ijerph17186726

**Published:** 2020-09-15

**Authors:** Boqiang Lin, Michael Adu Okyere

**Affiliations:** 1 School of Management, China Institute for Studies in Energy Policy, Collaborative Innovation Center for Energy Economics and Energy Policy, Xiamen University, Xiamen 361005, China; papaokyere70@gmail.com; 2 Belt and Road Research Institute, Xiamen University, Xiamen 361005, China

**Keywords:** multidimensional energy poverty, mental health, developing country

## Abstract

Regardless of growing interest and awareness of the effect of energy poverty on mental health, studies on this linkage have mainly relied on unidimensional measures of energy poverty with much concentration on advanced economies. Employing a two-wave socioeconomic survey, we analyzed the impact of multidimensional energy poverty on mental health in Ghana. We found energy poverty to heighten the chances of being mentally unhealthy. Using prices of liquefied petroleum gas and electricity as instruments for multidimensional energy poverty, we found that a rise in energy deprivation is associated with a 0.562-, 1.494- and 1.867-fold increase in the odds of being mildly, moderately and severely depressed, respectively. Among the indicators of multidimensional energy poverty, a deprivation in household appliance ownership (refrigerator ownership), recorded the highest impact on the depression levels of household heads. We concluded by urging policymakers to adopt a holistic approach in solving issues of energy poverty where simultaneous attention is given to all the dimensions of energy poverty since they collectively have detrimental effects on mental health, especially in a developing country setting.

## 1. Introduction

The United Nations Sustainable Development Goal (SDG) seven (SDG 7) stresses “access to affordable, reliable, sustainable, and modern energy for all” [1]. Globally, there has been about a 16% increment in electricity access between 1990 and 2016, as indicated by a United Nations (UN) report [2]. Despite this steady increase in access over the last few decades, the progress is skewed very much towards the global North and Asia, while the populace in sub-Saharan Africa (SSA) continues to live in darkness. In 2016, for example, over 600 million people in SSA lived without electricity, with about 80% residing in rural areas [3]. Globally, the World Health Organization (WHO) [4] estimates the loss of approximately 3.8 million lives annually from aliment owed to indoor air pollution attributable to the usage of traditional fuels such as wood and charcoal for heating and cooking. Again, a large proportion of these deaths prevail in SSA, as about 400 million people are estimated to continuously rely on biomass [4].

Nonetheless, there exists an inadequate understanding of the concept of energy poverty, particularly among decision-makers and politicians in SSA. Following Phoumin and Kimura [5], this stands to hamper the achievement of SDG 7. These policymakers generally focus on electrical grid connection or access to liquefied petroleum gas (LPG) as the only means of solving the issue of energy poverty and usually neglect elements of affordability such as end-usage. However, energy poverty is conceptualized as an idea that merges energy access and affordability, where lack of energy could impact economic and human development [6]. Thus, an energy-poor society stands to reel under fundamental challenges such as ill-health, poverty, illiteracy [7,8]. Hence there is a need to consider energy poverty as a multidimensional concept, as it is viewed by current studies [9,10].

Most recent scholarly that view energy poverty from a multidimensional perspective, also look at its effect on numerous development indicators such as health, poverty, education and the environment. Among these indicators, the relevant and forthright impact of energy poverty on health has fueled several recent studies—particularly in developing economies [6,11]. Despite this growing interest, little is known of the effect of energy poverty from a multidimensional framework on mental health, particularly in SSA. Mental illness in SSA is said to have increased the number of years lost to disability by about 52% between 2000 and 2015 [4]. This increasing trend is currently a cause of worry for policymakers, as it stands to negatively influence a range of outcomes such as lowering productivity levels, reducing life expectancy and deteriorating physical health [12].

Energy deprivation in itself, is also is evidenced as an primary source of cumulative stress or anxiety [13]. This is as a result of the discomfort it presents to households’ that are unable to access modern energy services. For example, most countries in SSA experience relatively high temperatures, with an average yearly temperature of about 46 °C [14]. This exposure to high temperatures has been noted to raise stress levels of people in this region, especially when residents are unable to properly cool their homes due to their inability to access adequate energy services. This disrupts sleep, causing crankiness, agitations and raising long-term anxiety levels [15]. As a result of this inadequate home energy, residents sometimes resort to coping strategies such as opening windows to deal with heat stress, which in some instances, ends up worsening their mental health conditions due to the fear of crime and violence [16,17].

Examining the impact of multidimensional energy poverty on the mental health of individuals in developing country settings is invaluable to influencing policies. Studies that have examined this relationship have all relied heavily on unidimensional measures of energy poverty and have mostly focused on industrialized economies [18,19,20,21]. This raises concerns about the universal applicability of their results [22] since their metrics of energy poverty are usually inadequate in tracking the progress towards SDG 7.

The problems mentioned above lead to an essential question: To what extent does energy poverty matter? This study, attempts to answer this research question by examining the effect of multidimensional energy poverty on mental health in a developing country. We explicitly expand the existing studies by adopting Nussbaumer et al.’s [9] framework in capturing energy poverty due to its ability to capture the deprivation in modern energy access, the intensity and incidence of energy poverty. Aside from estimating a fixed-effect model to address the issue of unobserved heterogeneity, we again demonstrate that our estimates are robust, as we deal with the issue of endogeneity that multidimensional energy poverty presented.

Unlike previous studies [18,19,20,21], this study focused on a developing economy, as suggested by Liddell and Guiney, [13]. We precisely employed the socioeconomic panel household survey on Ghana [23] in analyzing this relationship. Given the progress it has made over the last decade towards eradicating energy poverty, Ghana is a critical case study. In 2012, the country adopted a sustainable energy for all policy, with the sole purpose of accelerating progressive universal access to modern energy services. Effective strategies considered by this plan included the achievement of universal electricity accessibility by 2020. The policy targeted 50% access to LPG as well as introducing improved cookstoves to enhance the efficiency of biomass fuels. However, recent reviews in 2020 show that electricity access is far from its target (50% and 91% for rural and urban areas, respectively) [24], as biomass continues to be the primary source of cooking fuel for about 90% of rural folks [25].

Compared to the rest of the continent, Ghana has one of the highest rates of access to electricity, but is unlikely to achieve its target of attaining universal modern energy access in 2020 [26]. The cost of cooking fuel, inaccessible LPG service points, inadequate implementation framework of the LPG program [25], high levels of distribution and transmission loses and the low tariff structure crippling the financial stability of utility companies [26] are some of the reasons the target is likely to be missed. Despite possessing a relatively higher energy access rate on the continent, within the past decade, Ghana has experienced severe episodes of power crisis owing to dry periods and low water levels in its hydroelectric dam [27]. The recent—and perhaps most extended episode of this power crisis—occurred between 2012 and 2015. The situation led to a power rationing program, in which households were guaranteed between 12 and 13 h of electricity supply every 36 h [27]. This has raised concerns about the rate of energy deprivation and its rippling effect on the general economic and social wellbeing of households. It is imperative to identify the extent of energy poverty—especially from a multidimensional perspective in Ghana—as well as its impact on mental wellbeing to enhance the efficient formulation of policies targeted at vulnerable groups. Results from our study revealed that an increase in energy deprivation heightened one’s chances of being mentally unhealthy. Unlike previous studies [18,19,20,21], we found channels or indicators such as the lack of a refrigerator, electricity and modern cooking fuel inaccessibility, mobile phone deprivation and indoor air pollution to significantly deteriorate the mental health of household heads. We concluded by urging policymakers to adopt a holistic approach in solving issues of energy poverty where simultaneous attention is given to all the dimensions of energy poverty since they collectively have detrimental effects on mental health, especially in a developing country setting. We also advocate for subsidies on the end-use of energy, such as refrigerators, mobile phones for vulnerable groups.

The remainder of the study is arranged as follows: a review of the literature is presented in Section 2. Section 3 describes the methodology. Section 4 presents the results. Section 5 discusses and concludes the study.

## 2. Literature Review

### 2.1. Measuring Energy Poverty

Originally, the concept of energy poverty, as proposed by Lewis [28], was to show how insufficient energy usage affects livelihoods. Households who could not afford to maintain a comfortable indoor temperature were deemed to be energy-poor. Leach [29], among others, affirmed this definition of energy poverty by demonstrating how low-income households had a higher energy consumption per household income than their middle-income counterparts.

The 10% cutoff as an income-based measure of energy poverty was introduced by Boardman [30]. A household was classified as energy-deprived if it spent over 10% of its total income on energy. This indicator has been employed in several studies. In Scotland, for example, about 30% of households were identified as energy-poor when this measure was applied in 2015 [31]. Using household data between 1997 and 1998, Bennett et al. [32] employed this measure of energy poverty and found gas payment methods, income, type and composition of the household and state benefits to correlate with it. Papada and Kaliampakos, [33], Legendre and Ricci, [34], Scarpellini et al. [35] and O’Sullivan et al. [36] have all applied this measure. However, this threshold grew out of the UK’s experience, and issues about its universal applicability were always raised [22].

As an improvement to the 10% threshold approach, the low income high cost (LIHC) measure was suggested by Hills [37]. This metric captures the high overlapping energy cost against the low total income of the household. A household was classified to suffer from energy deprivation if its expenditure on energy was greater than the median energy expenditure, while the remainder of their equivalized income is lesser than the official poverty rate. The concept of minimum income standard (MIS) was also introduced by Moore [38]. This idea is defined as the minimum income that permits household members to determine the choices and opportunities that allowed them to integrate into society actively. Specifically, this measure categorized a household as energy-poor if its cost of energy was higher than its total income while accounting for its MIS and other housing costs.

The indicators mentioned above of energy poverty are mostly suitable in advanced economies where issues of energy deprivation are primarily centered on affordability. The situation in developing countries is more complicated as matters of energy poverty hover around affordability and accessibility [39]. This has made the provision of modern energy services critical to sustainable development goals due to its ability to motivate politicians in addressing the issue of energy poverty [1]. Besides, a modern energy services framework in looking at energy poverty gives way for a more explicit focus on the geographical dimensions of energy deprivation at the household level [39]. As a result, energy poverty is currently viewed from a multidimensional perspective by recent studies and notably among them is the International Energy Agency’s (IEA) energy development index (EDI). This index focuses on the national or regional adoption of modern fuel services [40]. The inability of this measure to capture energy deprivation at the household level forms its shortcoming [41,42]. Nussbaumer et al. [9] also proposed the multidimensional energy poverty index (MEPI). This index captures a household’s deprivation in modern energy access, together with the intensity and incidence of energy poverty.

Similarly, Sher et al. [43] and Ogwumike and Ozughalu [44] also contributed to other concepts of multidimensional energy poverty. The methodological reliability of this index is sometimes questionable, but can be useful if adequately developed [41,42]. It combines several indicators into a single measure of energy poverty for easy comparison across countries and periods. However, the justification for the inclusion of these indicators is mostly relative while the threshold that categorizes a household as energy-poor is set arbitrarily by the authors.

### 2.2. Energy Poverty and Mental Health

The broad range of developmental indicators affected by energy poverty has motivated existing research to measure energy poverty accurately. A summary of recent studies on the impact of energy poverty on health, environment, and the economy at large by Gonzalez-Eguino [45] concluded that the health impact of energy poverty was relevant and more straightforward. This is so because investments such as healthcare and access to clean energy are argued to replenish the health of individuals as their health deteriorates over time. Thus, a deprivation in energy affordability and accessibility worsens one’s health [46]. A WHO [4] report, for example, showed that most lives lost annually in poorer countries due to respiratory ailments were ascribed to indoor air pollution that emanated from the usage of traditional cooking fuels. Coupled with the fact that energy poverty is on the rise in many countries, this has sparked a rousing interest among researchers, consumer organizations and policymakers. For example, issues about energy poverty can be seen in several recent European Union (EU) policy proposals and regulatory documents [47].

Yet, despite this growing interest in mental health and energy poverty, relatively little is known about how energy deprivation influences mental wellbeing [18] in developing economies, particularly in SSA, as most of these studies [18,19,20,21] have been concentrated in advanced economies. For example, using energy efficiency investments in homes of low-income areas to represent energy poverty, Gray et al. [48] examined the linkages between energy poverty and the mental health of residents in a quasi-experimental field survey in Wales. Results from their study revealed that the energy efficiency investments had no significant impact on mental health. Employing a cross-sectional survey and using expenditure measures of energy poverty, Thomson et al. [20] and Rodriguez-Alvarez et al. [19] found a negative and significant relationship between energy poverty and mental wellbeing. However, their study failed to account for the unobserved characteristics of mental health since they used cross-sectional data. Employing panel data on 21 European countries, Welsch and Biermann [21] found that increases in energy prices were associated with a decline in mental wellbeing. They proxied energy prices for household energy poverty and concluded by suggesting the need for future studies to consider household measures of energy poverty that capture both the incidence and intensity to enable an in-depth analysis of the issue. With previous studies centered on European countries, Churchill et al. [18] examined this relationship using data from Australia. They also observed that energy poverty deteriorated the wellbeing of respondents.

Generally, it can be observed that recent studies [18,19,20,21] that have explored the energy-poverty and mental health nexus have relied on unidimensional measures of energy poverty with much focus on advanced economies [13]. These measures are argued to present a narrow overview of energy poverty and are usually inadequate for significant issues such as poverty and sustainable development [9]. Our study takes advantage of this gap and employs a multidimensional framework to establish the energy poverty-mental health relationship within a developing country context. This measure overcomes the limitations of the unidimensional indicators and produces an outcome that summarizes the information from the multiple indicators into a single indicator for a straightforward interpretation that can also be used to track progress in the SDGs.

## 3. Materials and Methods

### 3.1. Data

Data from a two-wave socioeconomic panel household survey on Ghana by the Economic Growth Center (EGC), based at Yale University and Institute of Statistical, Social and Economic Research (ISSER) at the University of Ghana [23] was employed for this study. It is a countrywide survey consisting of 5009 households from 334 enumeration areas (EAs) in Ghana to assist policymakers in formulating socioeconomic policies. The first and second waves of data collection were collected in 2010 and 2015, respectively. The survey covered a broader area of research than other nation-wide representative surveys in Ghana and other sub-Saharan countries. This survey was directed at the household head and other household members, where information about their demographics, energy services, as well as other socioeconomic characteristics, was gathered. Additionally, there was a section on the psychology of respondents, which covered issues on depression, personality and social networks. The analysis for this study was restricted to the household head since energy poverty was captured at the household level.

### 3.2. Measurement of Key Variables

#### 3.2.1. Mental Health

We used the Kessler psychological distress scale (K-10) to determine the mental health status of household heads [49]. This measure is used to identify an individual’s levels of depression or distress. With a five-level response scale, respondents scored ten questions regarding their emotional state (see Table A1 in the Appendix A). The score for each question ranged from 1—‘none of the time’ to 5—‘all of the time.’ We then summed the scores of the ten items, which yielded a minimum and maximum score of 10 and 50, respectively. Following Victoria (2002) [50], we categorized these scores as follows:

- For a score less than 20, the respondent is ranked to have low levels of depression;
- Between 20 and 24, the respondent is described to suffer from mild levels of depression;
- Between 25 and 29, the respondent is defined to be moderately depressed;
- For scores above 30, the respondent is described to be severely depressed.

#### 3.2.2. Energy Poverty

Capturing multiple dimensions of energy deprivation at the household level forms a significant contribution to this paper. To achieve this, we adopted Nussbaumer et al. [9] framework of the MEPI. Energy poverty under this framework is defined in terms of deprivation in modern energy services. The index fundamentally comprises of five dimensions of essential energy services that has six indicators (see Table 1). A household is classified as energy-poor if the amalgamation of the deprivations it faces surpasses a predefined cutoff. Energy for productive use and beyond household usage is not considered by this framework [51]. That notwithstanding, this index allows for decomposed analysis at a sub-national level, by region, by gender. End-usage such as appliance ownership that brings to the fore issues of affordability are also considered under this framework.

Mathematically, the dimensions of deprivation (d) across n households define the scope within which energy poverty is determined under this methodology. $Y=\left|y_{ij}\right|$ defines the n×d achievement of the $i^{th}$ household across j variables. For every row and column vector, $y_{i}=\left(y_{i1},y_{i2},\ldots ,y_{id}\right)$ and $y_{j}=\left(y_{j1},y_{j2},\ldots ,y_{jd}\right)$ represent the achievement of household i in the different variables and the distribution in the achievements of the variable j across all households, respectively. The weighting of the indicators is allowed under this framework, where a weighting vector (w) that encapsulates elements that correspond to the weight $\left(w_{j}\right)$ is applied to the variables (j).

The summation of this weight is defined as $\sum_{j=0}^{d}w_{j}=1$. Assuming $g=\left[g_{ij}\right]$ represents the deprivation matrix whose typical element $g_{ij}$ is defined by $g_{ij}=w_{j}$ when $y_{ij}<z_{j}$ and $g_{ij}=0$ when $y_{ij}\geq z_{j}$, where $z_{j}$ captures the deprivation cutoff in the variable j. Then, a column vector $C_{i}$ representing the deprivation score of the $i_{th}$ entry of deprivation is denoted by $C_{i}=\sum_{j=1}^{d}g_{ij}$. A predefined poverty threshold (k) is compared to the deprivation scores such that a multidimensionally poor household is determined if its deprivation score is at least greater or equal to 0.33 $\left(C_{i}\geq k\right)$, where k=0.33 [9].

In estimating a national household energy poverty index using the MEPI framework, the headcount ratio or incidence (H) is multiplied with the average intensity of energy poverty (A). The headcount ratio is expressed as H=qn, where, q is the total of the multidimensionally energy-poor households with n being the total number of households. Likewise, the expression for the average intensity of energy poverty (A) is given by A=∑i=1nCi(k)q.

#### 3.2.3. Other Control Variables

The study controlled for other attributes of the household that affects mental health theoretically and intuitively. These included the household head’s level of education, age, sex, body mass index (BMI), his/her health insurance status, monthly household expenditure, whether the household head smokes or not, place of residence and the region within which the household finds itself. A summary statistic of the variables employed for this study is presented in Table A2 of the Appendix A.

### 3.3. Econometric Strategy

The following equation is estimated in this study:

(1) $$Depression\_cat_{ijt}=\alpha_{s}+\beta_{i}MEPI_{it}+\gamma_{i}X_{it}+\varepsilon_{ijt}$$

where $Depression\_cat_{ijt}$ represents the $j^{th}$ category of household head i’s Kessler-10 scale for depression at time t; $MEPI_{it}$ captures the energy deprivation score for household i at time t; $X_{it}$ signifies a vector of characteristics of the household head including age, level of education, household income, etc.; β and γ represent the coefficient of MEPI and the vector of the control variables, respectively; ε represents the randomly distributed error term while $\alpha_{s}$ defines the household fixed effect.

Due to the categorical nature of our dependent variable (Kessler-10 depression measure), we used the logistic regression technique in estimating equation 1. This technique is argued to have more accurate predictions of the outcome variable than the ordinary least square (OLS) technique, especially when the dependent variable is categorical [52]. To be more specific, the multinomial logistic regression technique is adopted in this study since our outcome variable has more than two categories. However, the panel nature of our data raises issues about the unobserved heterogeneity; hence, a multinomial logistic regression with fixed effects (MLR-FE) other than the standard multinomial logistic regression (MLR) is preferred [53]. In addition to the MLR-FE controlling for the unobserved characteristics, the independence of irrelevant alternatives (IIA) assumption is also relaxed under this technique [54]. Nevertheless, the pooled MLR is presented in Table A3 of the Appendix A. The generalized structural equation modeling (GSEM) syntax in Stata is applied in estimating all the models in this study.

A likely potential source of endogeneity for energy deprivation is the omission of a variable. This could render the coefficient of energy deprivation either to be biased upwards or downward. We solve this using an instrumental variable MLR-FE, where electricity and liquefied petroleum gas (LPG) prices are used as instruments for energy poverty. The rationale behind this approach is that a rise in prices of LPG and electricity is related to the increase in energy deprivation, which turns to increase one’s chances of being mentally depressed. Findings from Narayan and Smyth, [55] and Fan and Hyndman, [56] backs to this linkage.

The propensity score matching (PSM) approach is also applied as an additional technique in addressing the endogeneity issue. This approach is often used in addressing problems of endogeneity in non-experimental surveys. It determines the average treatment effect (energy-deprived households in the case of this study) on our dependent variable (depression) [57,58].

## 4. Results and Discussion

### 4.1. Descriptive Statistics

**Figure 1:**
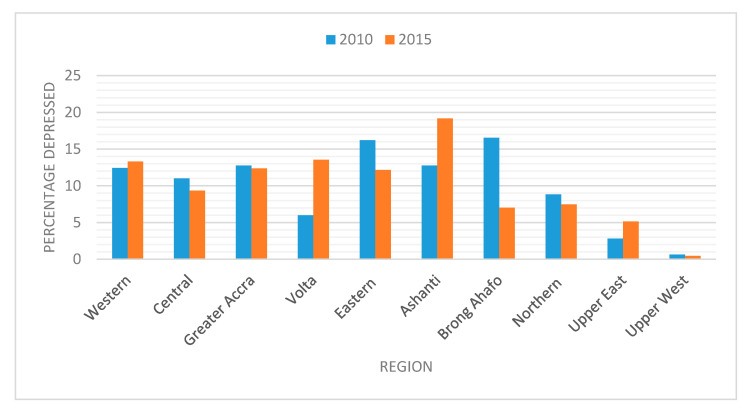
Percentage of depressed respondents between 2010 and 2015 by regions.

Figure 1 presents the percentage of respondents who suffered from depression (mildly, moderately and severely depressed) between 2010 and 2015 by regions. It can be observed that the Western, Volta, Ashanti and Upper East regions of Ghana recorded a rise in depression levels between 2010 and 2015. The people of the Volta region registered the highest increase in depression levels. Thus, between 2010 and 2015, depression levels in the Volta region rose by 8%, followed by Ashanti (6%), Upper East (2%) and Western (1%) region. On the other hand, the Central, Greater Accra Eastern, Northern and Upper West regions registered a decline in the percentage of depressed individuals. A significant reason for the decrease in depression levels in these regions was as a result of the basic needs model for mental health and development program, which provided services to people living with mental conditions in these areas back in 2010 [59].

### 4.2. Energy Deprivations in Ghana by the Various Multidimensional Energy Poverty Index (MEPI) Indicators between 2010 and 2015

The heights of deprivation on the chosen dimensions grounded on the threshold of multidimensional energy poverty during the study period are discussed in this section. The findings presented in Table 2 suggest a decline in the level of deprivation for all indicators between 2010 and 2015. Specifically, there was about an 11% reduction in the proportion of households that relied on biomass for cooking between 2010 and 2015. Efforts by the government to help reduce the overreliance on traditional cooking fuels via sensitization programs on the benefits of adopting modern energy services, particularly LPG [60], best explains this decline. However, a large percentage of households still depend on biomass than the other indicators of MEPI.

Access to electricity also witnessed improvements within the five-year period. Precisely, the proportion of households who were not connected to the grid decreased by 16%. Investments made in thermal and hydropower plants to augment the existing production is attributable to this decline [61]. Further, the telecommunication indicator witnessed a significant improvement, with about a 27% reduction in deprivation. This can be ascribed to the relative ease of transacting businesses that comes with mobile services such as mobile money transfers (MMT). This platform allows people to send, receive or store money onto their mobile devices, without requiring a bank account [62]. In Ghana, for example, this service witnessed about 216% increase in penetration as of 2015, while access to traditional banking services increased by just 2% in the same period [63].

### 4.3. The Multidimensional Energy Poverty Index (MEPI) in Ghana

Table 3 presents estimates for the intensity, incidence and the MEPI for Ghana between 2010 and 2015. A general decline can be observed among the indices. The headcount or incidence of multidimensional energy poverty decreased from 0.857 in 2010 to 0.755 in 2015. Thus, the proportion of households that were multidimensionally energy-poor, with a deprivation score at least equal to 0.330, decreased from 85.7% in 2010 to 75.5% in 2010. The average weighted deprivation of energy-poor households or intensity also declined by 9% between the period of study. Reduction in both intensity and incidence imperatively reflected a reduction in the MEPI. Thus, MEPI reduced from 0.497 in 2010 to 0.338 in 2015. This reduction in the MEPI is consistent with other studies in Ghana by [61,64].

**Figure 2:**
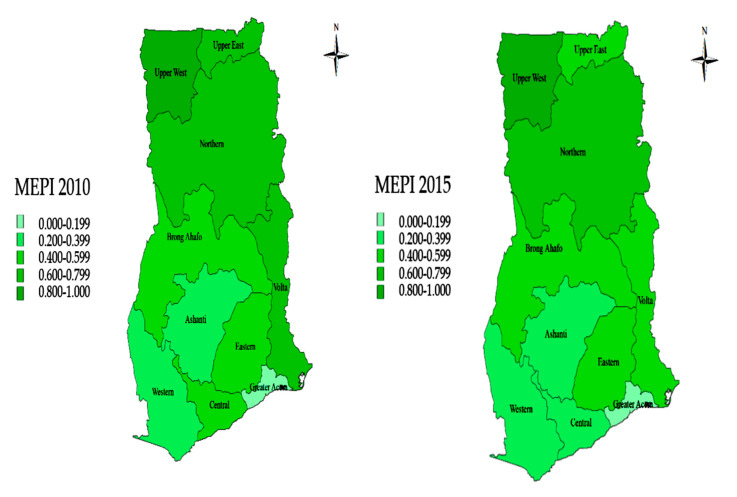
Regional decomposition of MEPI between 2010 and 2015.

A further analysis of the MEPI by regions is presented in Figure 2. Consistently, a decline in the MEPI can be observed across the ten administrative regions of Ghana for the understudied period. Regions in the northern part of Ghana recorded the highest rates of energy poverty among the other regions. In 2015, for example, energy poverty in the Upper West, Northern, and Upper East region was about 0.5812, 0.503 and 0.451, respectively, higher than the MEPI in the Greater Accra region (0.103). The prevalence of poverty in these regions could partly explain this trend [65], especially when income is argued to be a critical factor in modern cooking fuel adoption in Ghana [66]. Moreover, about 5% of the total LPG filling stations in Ghana is situated in these three regions [67], which therefore limits access to energy services in these areas.

### 4.4. Regression Results

Table 4 presents the odds for the relationship between energy deprivation and mental health in Ghana using the MLR-FE. As expected, the likelihood ratio (LR) test performed favored the fixed-effects (FE) model over the pooled model. The mean Variance Inflation Factor (VIF) also showed that multicollinearity was not present in the estimated model. Table 5 and Table 6 further presented estimates for the impact of energy poverty on mental health as we addressed the issue of endogeneity using the instrumental variable MLR-FE and propensity matching technique, respectively.

Compared to the reference group (i.e., low levels of depression), it can be observed from Table 4 that energy poverty has a positive relationship with all the categories of depression at a 1% significant level. In other words, the odds of being mentally unhealthy is more likely to increase as energy deprivation increases. Other covariates of influence in the model were education, sex of household head, household income, health insurance, smoking, body mass index (BMI) and region of residence, which are collectively consistent with the literature.

### 4.5. Dealing with Endogeneity

To be able to establish causality between energy poverty and depression, we presented a two-stage instrumental variable MLR-FE of the effect of MEPI on depression to deal with the endogeneity issue in Table 5. As an additional analysis to confirm that energy poverty deteriorates mental health, we again presented estimates using the PSM in Table 6. We also employed this same technique (PSM) in estimating the impact of the various indicators of MEPI on depression (Table 6).

Confirming our prior expectations, we find prices of electricity and LPG to be positively related to energy poverty from the first-stage model. Thus, a rise in the price of electricity and LPG increases energy deprivation. Consistent with the results in Table 4, we again found energy poverty to have a positive relationship with mental wellbeing after instrumenting MEPI with the prices of electricity and LPG. Compared to the baseline estimates (Table 4), we observed that endogeneity generated bias coefficients given that the IV estimates were comparatively smaller in magnitude. Therefore, holding all factors constant, a unit increase in energy deprivation is associated with 0.562, 1.494 and 1.867 increase in the odds of being mildly, moderately and severely depressed, respectively. The results from the PSM in Table 6 also suggest that households that were multidimensionally energy-poor on the average tend to have it heads suffering from relatively higher levels of depression compared to their counterparts who were not energy-poor. In general, it can be concluded from each of the approaches adopted in addressing the issues of endogeneity that energy poverty deteriorates mental health. This is so because energy deprivation in itself presents cumulative levels of stress or anxiety [13] due to the discomfort it presents households’ that are unable to access modern energy services.

Considering the impact of the respective indicators of energy poverty on depression, our results revealed that household heads who did not own a refrigerator were more susceptible to depression, followed by those who lacked access to electricity, mobile phone ownership, modern cooking fuel and indoor air pollution. The depression levels of household heads who did not own a refrigerator were about 1.218 higher than their counterparts who had one, at a 1% significance level. Individuals who are deprived of refrigerators are noted to experience frequent food spoilage, which, in turn, increase their depression levels [68]. In another breath, the lack of a refrigerator raises the anxiety levels, as those deprived of this indicator sometimes feel embarrassed to invite family or friends over for a visit. For a temperate country like Ghana with an annual average temperature ranging between 24 to 30 degrees Celsius [69], where the act of serving visitors with water (especially cold water) is deemed as a cultural good. One’s inability to offer his or her guest with cold water, as a result of not owning a refrigerator, brings him or her some level of embarrassment or discomfort and hence worsens their mental health status.

At a 1% significance level, the depression levels of respondents who were deprived of electricity were about 1.803 greater than their counterparts who had access. The lack of electricity access limits the activities people can enjoy. Recreational and relaxation activities such as watching TV or playing video games while enjoying the cool breeze from an air-conditioner or standing fan is cut off. This is noted to stimulate the anxiety levels of people [68]. Instead of finding healthy alternatives, people without electricity access sometimes slip into patterns of substance abuse if they have a history of substance abuse [70]. Moreover, the depression levels of people who lack mobile phones are about 0.891 higher than those who own one, at a 5% significance level. With the ease mobile money transfers has brought to the informal sector in Ghana [63], the anxiety levels of individuals who lack mobile phones are likely to rise due to the high transaction cost that comes with not having a phone. The lack of mobile phones also leads to social isolation as communication is cut off. Social isolation itself can serve as a stressor, particularly for vulnerable groups like the elderly and people with chronic conditions [71]. Household heads who experienced indoor air pollution were also observed to have a 0.769 depression level, higher than those who did not experience it, at a 5% significance level. The heightened inflammatory and neurodegenerative mediators that comes with cumulative biomass smoke inhalation [72] best explain this relationship. Thus, ultrafine carbon particles with a diameter of about 20–50 nm generated from the burning of biomass [73] find their way into the brain through the olfactory neurons or circulation passage through the blood–brain barrier and raise the anxiety levels of the individuals involved [74,75,76,77].

## 5. Conclusions

To the best of our knowledge, this is the first study of its kind in a sub-Saharan African nation like Ghana, that empirically investigates the effect of multidimensional energy poverty on mental health. The findings suggest a decline in energy deprivation for the understudied period. MEPI reduced from 0.497 in 2010 to 0.338 in 2015. This implies that MEPI reduced by 16% between 2010 and 2015 and that about 34% of households in Ghana were multidimensionally energy-poor in 2015. Furthermore, a large proportion of households were found to continuously use biomass for cooking and heating than the other indicators of MEPI. This is indicative that addressing problems concerning traditional sources and the use of fuel can substantially reduce energy poverty in Ghana. In addition, the decomposition of the MEPI by regions showed that households in Northern Ghana experienced a high prevalence of energy poverty. Consistent with previous studies [18,19,20,21], the key findings from this study suggest that energy poverty increases the odds of being mildly, moderately and severely depressed by 0.562, 1.494 and 1.867, respectively. However, unlike these studies that found channels such as the difficulty in maintaining adequate room temperature or purchasing prices of energy to relate negatively with mental wellbeing, our research found all the indicators MEPI except radio or television ownership to increase depression levels. Specifically, we observed that household heads who did not own a refrigerator experienced the highest impact of depression. The following policy implications are based on these findings.

- To reduce energy poverty and improve mental health, policymakers must consider a holistic approach in solving energy poverty where simultaneous attention is given to all of the dimensions of the MEPI;
- Although interventions such as the LPG program that sought to supply households with cookstoves and LPG cylinders have been implemented, the ability of beneficiaries to financially and sustainably use LPG cylinders and stoves continues to be a significant constraint [61,67]. Therefore, a mechanism that identifies and provides financial incentives to the beneficiaries of the program is recommended since higher prices of LPG was also found to increase depression through MEPI;
- Granting that the MEPI has reduced across all the ten regions of Ghana in the understudied period, there is still a high prevalence of it in the three Northern regions. A great deal of political weightiness is brought to the floor by this particular finding, as it tends to serve as a guide to politicians in targeting regions with of higher incidence of energy poverty;
- As energy poverty has been observed to deteriorate the mental health of respondents in this study. Policies makers must consider the role of access to clean, affordable and reliable energy play in mental health policies;
- Aside from concentrating on electricity access and modern fuel usage as means in reducing energy poverty, attention should be given to the end usage of energy (refrigerator and mobile phone ownership) as they play a vital role in the mental health status of people. We advocate for subsidies on these energy services for vulnerable groups.

### Limitation of the Study

Indicators such as the number of people living in the same household and its surface (in square-meter) have direct effects both on energy poverty and mental wellbeing, but due to data unavailability, they were not included in the analysis. Thus, future studies could look at this dimension.

## Figures and Tables

**Table 1:** Dimension and indicators of the Multidimensional Energy Poverty Index (MEPI).

Dimensions	Indicator (Weight)	Variable	Deprivation Cutoff (Poor If…)
Lightning	Electricity access (0.200)	Is a household connected to the national grid	False
Cooking	Modern cooking fuel access (0.200)	Biomass is the primary source of cooking fuel for the household	True
	Indoor air pollution (0.200)	Household employs biomass fuel in an enclosed room without chimney or window for cooking	True
Ownership of asset	Household appliance ownership of (0.130)	Owns a refrigerator or freezer	False
	Ownership of entertainment or education appliance (0.130)	Owns a radio or TV	False
Telecommunications	Means of telecommunication (0.130)	Owns a mobile phone	False

Source: Adopted from Nussbaumer et al. [9].

**Table 2:** Summary of deprivation indicators by energy services between 2010 and 2015.

Indicator	Weight	Deprived on Indicator (%)	
		2010	2015
*Lightning*			
Electricity access	0.200	46.631	30.954
*Cooking*			
Modern cooking fuel	0.200	88.727	77.735
Indoor air pollution	0.200	21.747	18.729
*Services provided using a household appliance*			
Household appliance ownership (refrigerator)	0.130	80.080	73.150
Entertainment/education appliance ownership (radio or television)	0.130	50.298	49.319
Telecommunication means (phone land line or mobile phone)	0.130	46.043	19.471

**Table 3:** Estimate for MEPI in Ghana between 2010 and 2015.

Index	2010	2015
Headcount/incidence (H)	0.857	0.755
Average intensity (A)	0.580	0.448
MEPI (H*A)	0.497	0.338

**Table 4:** Odds for the relationship between energy deprivation and depression in Ghana using multinomial logistic regression with fixed effects (MLR-FE).

Variables	Mildly Depressed	Moderately Depressed	Severely Depressed
MEPI	0.619 ***	1.582 ***	1.936 ***
	(0.237)	(0.413)	(0.524)
Education (base: none)			
Junior high school (JHS)	−0.360 ***	−0.162	−0.655 ***
	(0.109)	(0.162)	(0.220)
Senior high school (SHS)	−0.664 ***	−0.290	−0.095
	(0.187)	(0.289)	(0.329)
Tertiary	−0.506 ***	−0.553 *	−0.992 **
	(0.195)	(0.326)	(0.462)
Age of household head	0.004	0.004	−0.001
	(0.004)	(0.005)	(0.008)
Female	0.526 ***	0.679 ***	1.063 ***
	(0.113)	(0.165)	(0.226)
Ln (income)	−0.231 ***	−0.203 **	0.051
	(0.065)	(0.096)	(0.129)
Health insurance (yes)	−0.175 *	−0.345 **	0.020
	(0.106)	(0.156)	(0.226)
Smoking (yes)	0.117	0.547 **	0.997 ***
	(0.182)	(0.249)	(0.309)
Body mass index (BMI)	0.010	−0.202	0.162
	(0.148)	(0.181)	(0.296)
Urban	−0.172	−0.052	0.264
	(0.114)	(0.184)	(0.234)
Region (base: Western)			
Central	−0.459 **	−0.0361	0.546
	(0.207)	(0.287)	(0.387)
Greater Accra	−0.049	0.114	−0.460
	(0.186)	(0.302)	(0.471)
Volta	0.0681	0.264	0.136
	(0.218)	(0.348)	(0.530)
Eastern	0.0495	0.490*	0.924**
	(0.188)	(0.275)	(0.374)
Ashanti	−0.149	−0.123	0.047
	(0.174)	(0.284)	(0.390)
Brong Ahafo	0.265	0.089	0.254
	(0.185)	(0.304)	(0.406)
Northern	0.853 ***	1.315 ***	1.472 ***
	(0.234)	(0.317)	(0.470)
Upper East	0.719 ***	1.065 ***	0.866
	(0.274)	(0.396)	(0.619)
Upper West	0.621	1.244	−14.920 ***
	(0.705)	(0.856)	(0.531)
Constant	−0.457	−1.701 *	−5.589 ***
	(0.743)	(0.963)	(1.495)
LR test (FE vs pooled)	7.27 ***		
Log-likelihood	−2912.66		
McFadden’s pseudo R-squared	0.053		
VIF	1.43		
Observations	3754		

Robust standard errors in parentheses; *** *p* < 0.01, ** *p* < 0.05, * *p* < 0.1.

**Table 5:** Instrumental variable MLR-FE of the effect of MEPI on depression.

Variables	Mildly Depressed	Moderately Depressed	Severely Depressed
Panel A—Addressing the endogeneity of MEPI with prices of electricity and LPG			
MEPI	0.562 **	1.494 ***	1.867 ***
	(0.229)	(0.362)	(0.477)
Controls?	Yes	Yes	Yes
First stage			
Ln (electricity prices)	0.033 ***		
	(0.006)		
Ln (LPG prices)	0.014 **		
	(0.006)		

Note: All models in this table include all the control variables in Table 4. Standard errors in parentheses. *** *p* < 0.01, ** *p* < 0.05.

**Table 6:** PSM results of the effect of MEPI and its indicators on depression.

Variable	Average Treatment Effect on the Treated
MEPI	1.679 ***
	(0.385)
Indicators of MEPI	
Electricity access	1.083 ***
	(0.332)
Modern cooking fuel	0.844 *
	(0.479)
Indoor air pollution	0.769 **
	(0.325)
Refrigerator ownership	1.218 ***
	(0.437)
Radio/television (T.V) ownership	−0.0503
	(0.252)
Mobile phone ownership	0.891 **
	(0.364)

*** *p* < 0.01, ** *p* < 0.05, * *p* < 0.1.

## Appendix A

**Table A1:** Questions from the Kessler psychological distress scale (K10).

Please Indicate the Answer that Corresponds with You Experience in the Past 4 Weeks	None of the Time (Score 1)	A Little of the Time (Score 2)	Some of the Time (Score 3)	Most of the Time (Score 4)	All of the Time (Score 5)
About how often did you feel tired out for no good reason?					
About how often did you feel nervous?					
About how often did you feel so nervous that nothing could calm you down?					
About how often did you feel hopeless?					
About how often did you feel restless or fidgety?					
About how often did you feel so restless you could not sit still?					
About how often did you feel depressed?					
About how often did you feel that everything was an effort?					
About how often did you feel so sad that nothing could cheer you up?					
How many days were you unable to work?					

Source: Socioeconomic panel household survey in Ghana [23].

**Table A2:** Description and summary statistics of variables.

Variable	Description	Mean	SD
Depression	Kessler psychological distress scale (K10)	16.81	5.96
Energy poverty	Multidimensional energy poverty	0.44	0.25
Education	Highest Educational Level of Household head		
Junior high	Education (1 = junior high, 0 = other)	0.46	0.50
Senior high	Education (1 = senior high, 0 = other)	0.12	0.33
Tertiary	Education (1 = tertiary, 0 = other)	0.11	0.31
Age	Age of household head	44.76	13.62
Female	Sex of household head (1 = female and 0 = other)	0.31	0.46
Household income	Household monthly expenditure for both food and non-food items	440.77	438.7
Health insurance (yes)	Health insurance beneficiary	0.61	0.49
Smoking (yes)	Smoking habit	0.08	0.27
BMI (base: Normal)	Body Mass Index		
Underweight	BMI (1 = underweight, 0 = other)	0.03	0.05
Overweight	BMI (1 = overweight, 0 = other)	0.06	0.23
Obese	BMI (1 = obese, 0 = other)	0.93	0.24
Urban	Location (1 = urban, 0 = rural)	0.48	0.50
Region (base: western)	Region of residence		
Central	Region (1 = Central region, 0 = other)	0.11	0.31
Greater Accra	Region (1 = Greater Accra region, 0 = other)	0.17	0.38
Volta	Region (1 = Volta region, 0 = other)	0.08	0.26
Eastern	Region (1 = Eastern region, 0 = other)	0.12	0.33
Ashanti	Region (1 = Ashanti region, 0 = other)	0.17	0.38
Brong Ahafo	Region (1 = Brong Ahafo region, 0 = other)	0.11	0.31
Northern	Region (1 = Northern region, 0 = other)	0.06	0.22
Upper East	Region (1 = Upper East region, 0 = other)	0.03	0.16
Upper West	Region (1 = Upper West region, 0 = other)	0.01	0.07

**Table A3:** Effect of MEPI on depression from the pooled multinomial regression.

Variables	Mildly Depressed	Moderately Depressed	Severely Depressed
MEPI	0.572 **	1.532 ***	1.889 ***
	(0.221)	(0.387)	(0.504)
Controls?	Yes	Yes	Yes
Log-likelihood	−2916.338		
Pseudo R2	0.052		
Wald statistic	2137.72 ***		
Observations	3754		

*** *p* < 0.01, ** *p* < 0.05.
